# Early Seedling Growth Responses to *Gluconacetobacter diazotrophicus* Inoculation in Sugarcane: A Multivariate and Semi-Mechanistic Analysis

**DOI:** 10.3390/plants15192902

**Published:** 2026-09-23

**Authors:** Alex Guillen, Samuel Valle-Asan, Javier Barragan-Bustamante, Rafael Lazo-Sulca

**Affiliations:** Faculty of Science and Engineering, Universidad Estatal de Milagro UNEMI, Km. 1 ^1^/_2_ Via Virgen de Fátima, Milagro 091050, Guayas, Ecuadorrlazos@unemi.edu.ec (R.L.-S.)

**Keywords:** *diazotrophic* endophytes, plant–bacteria interaction, inoculum traceability, biological nitrogen fixation, root development, biomass allocation

## Abstract

*Gluconacetobacter diazotrophicus* is a sugarcane-associated diazotrophic endophyte whose functional relevance includes carbon oxidation, auxin-related signaling, colonization, and persistence. This study evaluated early sugarcane seedling responses to *G. diazotrophicus* inoculation under greenhouse conditions using phenotypic, multivariate, and semi-mechanistic analyses. The inoculum was supported by culture recovery, preservation continuity, and PCR-based molecular evidence, while seedling performance was assessed across eight treatments using shoot length, root length, net gains, total biomass, and root-to-shoot allocation. Responses were treatment-dependent rather than uniformly growth-promoting. The non-inoculated water control T8 reached the highest absolute endpoint performance, whereas T2 was the strongest inoculated treatment, showing the most balanced profile across shoot gain, root gain, and total biomass. Treatment contrasts separated absolute control performance from inoculated-treatment ranking. Multivariate profiling confirmed strong treatment separation (PERMANOVA: pseudo-*F* = 114.07, *R*^2^ = 0.700, *p* = 0.001), and the standardized fingerprint showed that greater inoculum complexity did not necessarily translate into stronger plant response. The semi-mechanistic model explained 72.8% of total biomass variation, with acceptable collinearity (maximum *VIF* = 2.77). Overall, early seedling performance was structured by exposure, inoculum architecture, and phenotypic expression rather than inoculation presence alone.

## 1. Introduction

Sugarcane is a crop whose early establishment depends strongly on the successful integration of root development, shoot expansion, and nutrient acquisition during the initial stages of seedling growth. In this context, plant-associated diazotrophic bacteria have attracted particular interest because they may enhance early vigor through mechanisms that extend beyond simple nutrient supplementation, including endophytic persistence, developmental signaling, and metabolic interaction with sugar-rich plant tissues [1,2]. Among these microorganisms, *G. diazotrophicus* has a distinctive biological relevance because it was originally described as an acid-tolerant nitrogen-fixing bacterium associated with sugarcane and later consolidated as a species closely adapted to this host environment [3,4]. Its association with sugarcane is especially important because this bacterium is not merely rhizosphere-associated; it can occupy internal plant compartments and persist in tissue environments characterized by high sucrose availability and low pH, conditions that are biologically compatible with its endophytic lifestyle [5,6].

The functional interest in *G. diazotrophicus* derives from the fact that its plant-associated performance is supported by a metabolic repertoire broader than nitrogen fixation alone. Genome-scale analysis of strain Pal5 revealed genes linked to nitrogen fixation, sugar metabolism, transport systems, exopolysaccharide synthesis, acid adaptation, and plant growth-related functions, indicating that this species is metabolically equipped to operate in close association with plant tissues rather than as a strictly free-living diazotroph [7]. This interpretation is consistent with experimental studies showing that *G. diazotrophicus* uses constitutive oxidative metabolism and extracellular carbohydrate-processing traits that are compatible with sugarcane-associated niches. In particular, the structural levansucrase gene *lsdA* has been shown to be a conserved and functional locus in the species, supporting sucrose conversion into oligofructans and levan [8]. In addition, phosphate-dependent enhancement of levan production has been demonstrated in strain Pal5, reinforcing the importance of extracellular fructan synthesis as part of the organism’s plant-associated metabolic behavior [9]. Oxidative metabolism is also a defining feature of the species: a membrane-bound pyrroloquinoline quinone (PQQ)-dependent alcohol dehydrogenase system has been characterized in *G. diazotrophicus*, supporting the broader concept of a periplasmic oxidative metabolism typical of acetic acid bacteria and relevant to carbon processing under plant-associated conditions [10]. Although the present study does not directly quantify fluxes through these pathways, the available literature clearly supports the interpretation of *G. diazotrophicus* as a bacterium metabolically adapted to a sucrose-rich endophytic niche [7,8,9,10].

A second key branch of its functional relevance is diazotrophy. Biological nitrogen fixation has long been recognized as one of the defining traits of sugarcane-associated *G. diazotrophicus*, and its ecological value is reinforced by the fact that the bacterium can function under microaerobic conditions compatible with internal plant tissues [5,11]. More recently, high-resolution fitness analysis under diazotrophic growth confirmed the importance of genes related to the nitrogenase machinery, electron transfer, and nitrogen assimilation, strengthening the view that nitrogen fixation in this species is a central but tightly regulated process rather than a generic background trait [11]. This matters for the present work because early seedling vigor may reflect not only the presence of a bacterium with diazotrophic potential, but also the integration of that potential with carbon metabolism, host compatibility, and treatment-dependent inoculum behavior.

A third functional branch involves auxin-related signaling, which provides a plausible link between microbial metabolism and root developmental outcomes. In *G. diazotrophicus*, auxin biosynthesis has been repeatedly associated with plant growth-related effects. Earlier work showed that strains defective in cytochrome *c* biogenesis were also impaired in indole-3-acetic acid biosynthesis, linking respiratory/metabolic competence with auxin production [12]. Later, transposon mutagenesis identified genes associated with indole-3-acetic acid biosynthesis in strain Pal5 and supported a tryptophan-dependent route connected with the indole-3-pyruvate branch, thereby providing a more direct molecular basis for auxin-related signaling in this species [13]. This branch is especially relevant for a seedling-based study because early responses in root architecture, net root gain, and biomass partitioning are precisely the type of outputs that may emerge from microbe-modulated auxin dynamics even when nitrogen fixation is not the only active process [12,13].

A fourth functional branch concerns colonization, persistence, and biofilm-related stability. Plant growth promotion by endophytes depends not only on metabolic potential, but also on the ability to adhere, persist, and remain functionally active within the host environment. In *G. diazotrophicus*, exopolysaccharide production has been shown to be required for biofilm formation and plant colonization, with *gumD*-associated disruption leading to defective adhesion and persistence traits [14]. Additional work has shown that intracellular polyphosphate levels influence abiotic stress tolerance and biofilm formation in this species, indicating that persistence is supported by a broader physiological framework rather than by exopolysaccharides alone [15]. Together, these findings justify treating colonization as a mechanistically distinct branch in the conceptual model of the study.

Under this theoretical framework, Figure 1 summarizes the metabolic-functional rationale used to interpret early sugarcane seedling responses to the validated *G. diazotrophicus* inoculum. The figure is intended as a literature-grounded conceptual framework, not as direct experimental evidence of each metabolic pathway.

Despite the well-established biological relevance of *G. diazotrophicus*, an important analytical gap remains. Many studies have documented colonization, nitrogen fixation, or growth-promotion effects, yet fewer have evaluated early plant responses as a multidimensional response architecture integrating inoculum validation, exposure structure, phenotypic expression, and explanatory modeling. This gap is relevant because early seedling performance is not normally expressed as a single-variable response. Instead, it emerges through coordinated variation in shoot elongation, root expansion, integrated biomass, and allocation patterns. A multivariate and semi-mechanistic framework is therefore particularly suitable for this system because it allows the plant response to be interpreted as the result of interacting functional dimensions rather than as an isolated endpoint effect.

Accordingly, this study aimed to evaluate early sugarcane seedling responses to *G. diazotrophicus* inoculation under greenhouse conditions by integrating inoculum validation, phenotypic assessment, treatment contrasts, multivariate profiling, and biomass modelling. Specifically, we examined whether treatment-dependent differences in shoot growth, root growth, biomass accumulation, and root-to-shoot allocation could be resolved as a coherent early-response profile.

## 2. Results

### 2.1. Phenotypic Validation and Operational Preservation of the Inoculum

Before plant inoculation, the microbial material used in this study corresponded to a CINCAE-preserved sugarcane-associated isolate previously assigned to *G. diazotrophicus* based on institutional sequence-based identification. The sequencing report is retained in the CINCAE institutional records. In the present experiment, the isolate showed recoverable growth on LGI-P semisolid medium, LGI-P solid medium, and PDA, together with Gram-negative staining compatible with the expected bacterial profile. These observations were interpreted as pre-inoculation evidence of viability, purity support, and handling continuity, rather than as the sole basis for taxonomic assignment. The inoculum was standardized before application to an OD600 of 0.82 ± 0.04, corresponding to 1.35 × 10^8^ ± 0.21 × 10^8^ CFU mL^−1^. These values confirmed consistent inoculum density across inoculated treatments. The culture recovery and preservation evidence supporting this pre-inoculation validation workflow is summarized in Figure 2.

### 2.2. Molecular Confirmation of the Inoculum by PCR Amplicon Profiles

The molecular validation of the inoculum was performed using three PCR amplicon profiles selected to provide complementary support for bacterial identity and *G. diazotrophicus*-associated detection. Figure 3 shows three PCR amplicon profiles used as molecular support for the inoculum. The 218 bp product corresponded to a *G. diazotrophicus* associated 16S–23S rDNA ITS/intergenic spacer marker and was the most specific molecular layer used in this study. The approximately 1491 bp product corresponded to a nearly full-length bacterial 16S rRNA marker, whereas the 395 bp product corresponded to a partial bacterial 16S rRNA fragment. Therefore, the 1491 bp and 395 bp products supported bacterial identity at marker level but were not species-specific. Importantly, the 395 bp product was not interpreted as nifH, and none of the PCR profiles was treated as sequencing-based taxonomic confirmation. The primer pairs, target regions, expected amplicon sizes, and interpretive scope of each PCR assay are detailed in Section 4.3.

The PCR results were interpreted as experimental verification of inoculum identity and handling continuity within the present greenhouse assay. Taxonomic assignment to *G. diazotrophicus* was not based solely on band presence, but on the prior CINCAE sequence-based identification of the isolate, complemented here by reproducible amplification of the expected marker profiles. The 218 bp 16S–23S rDNA ITS/intergenic spacer product provided the most specific marker-level support for the target species, whereas the approximately 1491 bp and 395 bp 16S rRNA products provided broader bacterial identity support. The 395 bp amplicon was considered a partial 16S rRNA marker and was not interpreted as nifH amplification or direct evidence of nitrogenase activity. The lane labelled Cp in the gel layout was not considered as supporting evidence because no clear positive-control band was visible in the displayed image; therefore, the PCR interpretation was based on the reproducible sample amplicon profiles, the negative extraction controls, and the prior CINCAE sequence-based identification.

The convergence between culture recovery, Gram-negative staining, and reproducible PCR banding patterns strengthened the operational validation of the microbial inoculum before plant application. This validation step was essential to ensure that the subsequent plant-growth responses were evaluated in relation to a microbiologically supported inoculum rather than to an undefined microbial preparation.

### 2.3. Phenotypic Response from Baseline to Final Evaluation

All treatments exhibited measurable growth by day 30, but the magnitude of the response differed strongly among treatments (Figure 4). Final shoot length differed significantly among treatments according to Welch’s ANOVA (*p* = 1.92 × 10^−33^). T8 showed the highest mean final shoot length (53.12 mm), followed by T2 (47.46 mm), whereas T3 and T7 remained among the lowest groups, with mean final shoot lengths of 30.82 mm and 30.85 mm, respectively. The same treatment-dependent pattern was observed for net shoot gain (Welch *p* = 3.36 × 10^−33^), where T8 reached the highest mean gain (42.15 mm), followed by T2 (36.33 mm).

Root development also showed strong treatment dependence. Final root length differed significantly among treatments (Welch *p* = 3.86 × 10^−19^), with T2 and T8 showing the highest mean values, 100.12 mm and 99.25 mm, respectively. Net root gain followed the same pattern (Welch *p* = 8.45 × 10^−19^), with T2 and T8 reaching nearly equivalent mean gains of 85.29 mm and 84.41 mm. This indicates that T2 was the strongest inoculated treatment for root development, whereas T8 represented the strongest non-inoculated endpoint.

Total biomass differed significantly among treatments (Welch *p* = 7.82 × 10^−53^). T8 showed the highest mean total biomass (1184.37 mg), followed by T2 (993.41 mg). In contrast, T7 showed the lowest mean biomass (295.18 mg), indicating that the rhizospheric consortium with greater structural complexity did not translate into superior biomass accumulation under the evaluated early-seedling conditions. Root-to-shoot allocation also differed significantly among treatments (Welch *p* = 1.49 × 10^−16^), but elevated allocation ratios did not necessarily correspond to higher total biomass. This confirms that allocation pattern and integrated biomass response should be interpreted as related but non-equivalent outputs.

### 2.4. Treatment-Level Contrasts Separating Absolute Endpoint Performance from Inoculated-Treatment Ranking

To clarify the treatment structure observed in the distributional analysis, treatment-level contrasts were performed to separate two different biological questions: first, which treatment showed the strongest absolute endpoint performance; and second, which inoculated treatment showed the strongest response within the inoculated subset. This distinction was necessary because T8 corresponded to the non-inoculated water control, whereas T1–T7 corresponded to inoculated treatments with different isolate configurations.

When total biomass was contrasted against T8, all inoculated treatments showed lower biomass accumulation than the non-inoculated control (Figure 5A). The smallest biomass deficit relative to T8 was observed for T2, with a mean difference of −190.96 mg, whereas the largest deficit was observed for T7, with a mean difference of −889.19 mg. The remaining inoculated treatments also showed marked biomass reductions relative to T8, ranging from −493.38 mg in T1 to −660.35 mg in T6.

Shoot gain followed the same general contrast pattern (Figure 5B). All inoculated treatments showed lower shoot gain than T8. T2 again showed the smallest deficit relative to T8, with a mean difference of −5.82 mm, whereas T3 and T7 showed the largest reductions, with mean differences of −22.37 mm and −22.35 mm, respectively.

Root gain showed a more differentiated response (Figure 5C). Unlike biomass and shoot gain, T2 did not differ significantly from T8 for root gain, with a mean difference of only +0.87 mm. This indicates that T2 reached a root-growth response statistically comparable to the non-inoculated control. In contrast, the other inoculated treatments showed lower root gain than T8, with the strongest reduction observed in T7 (−48.69 mm).

To rank the inoculated treatments independently from the non-inoculated control, total biomass was then contrasted using T2 as the inoculated reference treatment (Figure 5D). All other inoculated treatments produced significantly lower biomass than T2. The closest inoculated treatment was T1, with a mean difference of −302.42 mg relative to T2, whereas T7 showed the largest biomass reduction, with a mean difference of −698.23 mg. These contrasts support the classification of T2 as the strongest inoculated treatment, while T7 represented the weakest inoculated biomass profile despite its greater consortium complexity.

These contrasts identified T8 as the strongest absolute endpoint and T2 as the leading inoculated treatment across the evaluated growth traits.

### 2.5. Multivariate Treatment Fingerprint and PERMANOVA Support

The integrated multivariate analysis was used to determine whether the evaluated treatments produced distinguishable response fingerprints when exposure, inoculum structure, phenotypic response, and final outcome variables were analyzed jointly (Figure 6). The PCA was performed using the standardized matrix composed of OD600, strain count, colonization potential, dose plausibility, shoot gain, root gain, total biomass, and root-to-shoot ratio. The first two principal components explained 76.5% of the total multivariate variance, with PC1 accounting for 56.2% and PC2 for 20.3% of the variation.

The PCA biplot showed that the treatments did not occupy a single common response space (Figure 6A). T8 was positioned apart from the inoculated treatments and aligned with stronger shoot gain, root gain, and total biomass vectors.

Among the inoculated treatments, T2 occupied the closest position to the high-performance region of the PCA space. This agrees with its stronger inoculated profile in the previous analyses, particularly for root gain, shoot gain, and total biomass. In contrast, T7 was displaced toward the region associated with exposure and structural descriptors rather than with biomass accumulation. This indicates that greater inoculum structural complexity did not necessarily translate into superior early seedling performance under the evaluated conditions.

The standardized treatment fingerprint heatmap provided the clearest treatment-by-variable interpretation of the multivariate structure (Figure 6B). T2 showed the most balanced inoculated profile, with positive standardized values for OD600 (+1.5), colonization potential (+0.7), dose plausibility (+1.0), shoot gain (+0.8), root gain (+0.8), total biomass (+0.9), and root-to-shoot ratio (+0.5). This pattern indicated a balanced inoculated profile across exposure, phenotypic, and outcome descriptors.

T8 showed the opposite analytical architecture. As expected for the non-inoculated control, it had negative standardized values for inoculum-related descriptors, including OD600 (−1.8), strain count (−1.5), colonization potential (−2.4), and dose plausibility (−1.9). However, it showed the strongest outcome-oriented profile, with positive standardized values for shoot gain (+1.3), root gain (+0.8), and total biomass (+1.5).

T7 showed a contrasting pattern. It had high standardized values for strain count (+2.3), colonization potential (+1.3), and dose plausibility (+1.7), but negative values for shoot gain (−0.7), root gain (−1.0), and total biomass (−1.1).

The remaining inoculated treatments showed intermediate or mixed profiles, confirming that the inoculated subset was not phenotypically uniform. T4 and T5 showed moderate or mixed values, while T3 and T6 tended to show negative standardized values across several phenotypic or outcome descriptors, confirming that the inoculated subset was not phenotypically uniform. Instead, each treatment expressed a distinct combination of exposure, structure, phenotype, and outcome signals.

The core-level contribution panel summarized these treatment-specific patterns into four response-oriented analytical dimensions: exposure, structure, phenotypic performance, and outcome (Figure 6C). This panel should be interpreted as a normalized analytical contribution profile, not as microbial abundance. Validation descriptors were deliberately excluded from this PCA–PERMANOVA matrix because identity support and molecular validation were used to establish inoculum traceability, not to drive treatment separation in the multivariate response space. Therefore, Figure 6C shows how exposure, inoculum structure, seedling phenotype, and final biomass contributed differently across treatments, supporting the interpretation that the evaluated system contained multiple response architectures rather than a single uniform inoculation response.

PERMANOVA confirmed that treatment identity explained a large and statistically significant proportion of the integrated multivariate variation (Pseudo-*F* = 114.07; *R*^2^ = 0.700; *p* = 0.001; 999 permutations; Figure 6D). Thus, approximately 70% of the multivariate structure was attributable to treatment differences. Because the dispersion panel also showed differences in within-treatment spread, the PERMANOVA result was interpreted together with multivariate dispersion rather than as a location-only effect. Overall, PERMANOVA therefore supported significant treatment-level separation in the standardized multivariate matrix.

### 2.6. Inoculated-Treatment Ranking and Decoupling Between Exposure and Performance

To avoid interpreting the non-inoculated control as an inoculation response, the inoculated treatments were further examined as an independent subset (T1–T7; Figure 7). This analysis focused on the internal ranking of inoculated treatments and on the relationship between exposure intensity and phenotypic performance. The standardized profile was calculated within the inoculated subset, using shoot gain, root gain, total biomass, and root-to-shoot ratio as the main performance descriptors.

The z-score profile showed that T2 was the only inoculated treatment with consistently positive standardized values for shoot gain (+1.86), root gain (+1.51), and total biomass (+1.84) (Figure 7A). T1 showed a positive biomass signal (+0.41) and the highest root-to-shoot allocation signal (+1.23), but its root gain was slightly below the inoculated-treatment mean (−0.24). T5 showed a positive root-gain signal (+0.73) but only a weak positive biomass signal (+0.07), whereas T4 showed moderate root gain (+0.50) but a slightly negative biomass signal (−0.26). In contrast, T7 showed negative values for shoot gain (−1.03), root gain (−1.55), and total biomass (−1.47), despite its high inoculum complexity.

The ranking analysis confirmed the same pattern (Figure 7B). T2 ranked first among inoculated treatments for shoot gain, root gain, and total biomass, indicating that it was not favored by a single isolated trait but by a coordinated performance profile. T1 ranked second for shoot gain and total biomass, but only fourth for root gain. T5 ranked second for root gain and third for total biomass, whereas T7 ranked last for root gain and total biomass. Therefore, the inoculated subset did not behave as a uniform group, and the strongest inoculated response was clearly concentrated in T2.

The OD600–biomass relationship further showed that exposure intensity was not a monotonic proxy for plant performance (Figure 7C). T2 combined the highest OD600 among inoculated treatments (0.18) with the highest inoculated mean biomass (993.41 mg). However, T7 also showed a relatively high OD600 value (0.16) but the lowest inoculated biomass response (295.18 mg). These results indicate that OD600 alone did not explain treatment performance and should be interpreted only as an exposure descriptor within the broader treatment architecture.

To summarize inoculated performance across the main growth outputs, a response balance index was calculated from min–max normalized shoot gain, root gain, and total biomass within the inoculated subset (Figure 7D). T2 reached the maximum balance index value (1.00), followed by T1 (0.51), T5 (0.48), and T4 (0.47). T7 had the lowest value (0.00), confirming that the six-isolate rhizospheric consortium did not translate its greater structural complexity into a superior early-seedling response. This index confirmed T2 as the most balanced profile within the inoculated subset.

### 2.7. Semi-Mechanistic Biomass Model

To integrate the experimental evidence into a single explanatory structure, a conservative semi-mechanistic model was fitted using total biomass as the response variable and four standardized predictor cores: validation, exposure, inoculum structure, and phenotypic performance. Unlike the PCA–PERMANOVA matrix used in Figure 6, the validation core was retained here only as an inoculum-traceability component. Therefore, it was not used to separate treatments in multivariate response space, but to test whether biomass variation could be interpreted after accounting for the experimentally verified identity and molecular support of the inoculum. The model was used as an explanatory framework to test whether final biomass could be explained by an ordered set of experimentally traceable components: inoculum validation, exposure intensity, inoculum architecture, and measurable early seedling growth.

The core-level semi-mechanistic model explained a substantial proportion of the variation in total biomass response (*R*^2^ = 0.728; adjusted *R*^2^ = 0.725; *RMSE* = 177.19 mg; *n* = 350), indicating that the evaluated analytical cores captured the main structure of the treatment-response system. As shown in Table 1 and Figure 8B, the phenotypic core was the strongest positive predictor of total biomass (β = 0.546, 95% CI: 0.478 to 0.613, *p* < 0.001), confirming that shoot and root gain were the main direct contributors to biomass accumulation. The exposure core also showed a positive effect (β = 0.239, 95% CI: 0.145 to 0.333, *p* < 0.001), supporting the relevance of inoculum exposure intensity and dose plausibility as upstream contributors to plant response.

In contrast, the validation core and structure core showed negative standardized coefficients (β = −0.307 and β = −0.310, respectively; both *p* < 0.001). These negative coefficients should not be interpreted as evidence that isolate validation or strain structure reduced plant performance directly. Instead, they indicate that, after accounting for exposure and phenotypic response, these cores captured treatment-level contrasts associated with inoculated profiles that did not necessarily exceed the strongest non-inoculated endpoint response. Collinearity diagnostics supported the stability of the model, with all VIF values below 3.0 and a maximum VIF of 2.77. Therefore, the model supports a structured interpretation in which total biomass was primarily explained by phenotypic growth response, with additional contribution from inoculum exposure, while validation and strain-structure descriptors acted mainly as traceability and treatment-composition controls.

Figure 8A shows the core-level association structure supporting the model. The network indicates that validation, exposure, structure, phenotype, and biomass were not isolated analytical components, but interconnected layers of the experimental system. Figure 8B provides the most important inferential result of this model. The phenotypic core had a positive standardized contribution to biomass, confirming that shoot gain, root gain, and root-to-shoot allocation captured biologically relevant variation in seedling performance. The exposure core was also positive, indicating that OD600-associated exposure and dose plausibility contributed to biomass variation. In contrast, the structure core showed a negative conditional coefficient, meaning that increasing the number of isolate components did not produce a proportional increase in biomass. Thus, structure did not show a proportional positive association with biomass after adjustment for the other cores.

The validation core also showed a negative conditional coefficient after adjustment for exposure, structure, and phenotype. This result should not be interpreted as evidence that validation is biologically detrimental. Rather, it reflects the structure of the dataset, where the non-inoculated control reached high absolute biomass while having no inoculum validation score. Therefore, the coefficient indicates that verified inoculum identity was necessary for biological traceability, but not sufficient by itself to guarantee higher biomass. This interpretation is consistent with the broader treatment response pattern, where T2 represented the most balanced inoculated treatment, whereas T8 reached the highest absolute endpoint biomass as the water control.

The diagnostic panels further supported the stability of the model. The observed-versus-predicted plot showed a clear positive fit between measured and predicted biomass, while the Q–Q plot and residual histogram indicated acceptable residual behavior for an explanatory greenhouse model. The residual-versus-fitted plot did not indicate a collapse of the model structure, and the VIF diagnostics remained below the threshold of 5. Cook’s distance values did not show a dominant influential observation capable of driving the overall interpretation. Overall, the semi-mechanistic model supports a cautious but useful conclusion: early sugarcane seedling biomass under the evaluated *G. diazotrophicus* framework was structured by the interaction among exposure, inoculum architecture, and phenotypic expression, rather than by inoculation presence alone.

## 3. Discussion

### 3.1. Early Seedling Responses Are Better Interpreted as an Integrated Response Architecture than as Isolated Trait Shifts

The present study shows that early sugarcane seedling responses to *G. diazotrophicus* are more accurately interpreted as a multidimensional response architecture than as a set of independent changes in isolated variables. This point is important because the experimental signal did not emerge as a uniform increase across all endpoints, but rather as a structured reorganization of shoot gain, root gain, total biomass, and root-to-shoot allocation. Such behavior is consistent with recent multivariate phenotyping approaches, in which early plant plasticity is understood as a coordinated system of morphophysiological adjustments rather than as a unidimensional response [16].

### 3.2. Why the Non-Inoculated Control Showed the Highest Absolute Biomass

The higher absolute biomass observed in the non-inoculated water control should not be interpreted as evidence against the biological relevance of *G. diazotrophicus*. Instead, it indicates that, under the evaluated greenhouse conditions and within a 30-day seedling window, baseline plant growth without microbial exposure represented the strongest absolute endpoint. A plausible explanation is that inoculated seedlings initially allocated part of their carbon and physiological resources to plant–microbe interaction processes, including bacterial adhesion, colonization, extracellular polymer-mediated persistence, and hormone-related developmental adjustment, rather than translating the interaction immediately into greater total biomass. This interpretation is consistent with the known biology of *G. diazotrophicus*, whose plant-associated performance involves not only diazotrophy but also sucrose-linked carbon metabolism, oxidative metabolism, auxin-related signaling, and colonization/persistence traits [7,8,9,10,11,12,13,14,15]. Therefore, the absence of biomass superiority over T8 does not exclude functional inoculation effects; rather, it suggests that these effects were treatment-specific and trait-dependent. Within this framework, the inoculated response should be interpreted as trait-dependent rather than as a uniform biomass advantage. Because the present study did not directly quantify *in planta* nitrogen fixation, nitrogenase activity, bacterial tissue density, or phytohormone concentrations, the proposed mechanism should be considered a literature-supported interpretation rather than direct biochemical proof.

### 3.3. The Biological Plausibility of the Observed Response Is Consistent with the Known Functional Behavior of Gluconacetobacter diazotrophicus

The response patterns documented here are biologically plausible in light of the established literature on diazotrophic endophytes and sugarcane-associated *G. diazotrophicus*. Earlier studies on biological nitrogen fixation in non-legume systems established that plant growth promotion by diazotrophs should not be interpreted exclusively through nitrogen input, but also through broader physiological interactions between plant and microbe [17]. This broader view is especially relevant in sugarcane, where *G. diazotrophicus* has been described as a bacterium with a specific ecological association with the host rather than as a generic free-living inoculant [18].

The developmental side of this interaction is also supported by evidence that diazotrophic endophytes, including *G. diazotrophicus*, may produce indole-related compounds and gibberellins under defined conditions, thereby contributing to plant growth through hormone-linked effects in addition to nitrogen-related functions [19]. This supports the interpretation that early seedling responses may involve integrated nutritional and signaling processes rather than biomass accumulation alone [17,18,19].

### 3.4. Root-Associated Responses and Biomass Integration Are Compatible with Context-Dependent Colonization and Diazotrophic Performance

The present results indicate that the biological response of sugarcane seedlings depended on more than inoculum presence alone. This is consistent with earlier evidence showing that colonization by *G. diazotrophicus* is sensitive to plant physiological context. For example, colonization has been reported to decrease under high nitrogen supply [20], and the benefit to sugarcane growth has been shown to differ when wild-type diazotrophic strains are compared with Nif-deficient mutants [21]. In addition, population dynamics studies demonstrated that bacterial performance and plant response vary among sugarcane cultivars, indicating that the host background participates actively in shaping the outcome of the association [22].

Within that context, the root-sensitive behavior observed here is biologically meaningful. Root gain and allocation patterns were among the variables that contributed to treatment discrimination, even when they were not always aligned with the highest final biomass values. This suggests that early inoculation effects may first emerge as developmental reconfiguration rather than as a simple increase in final size. That interpretation is also compatible with evidence that auxin biosynthesis in *G. diazotrophicus* is functionally linked to bacterial physiological competence; mutants impaired in cytochrome *c* biogenesis are also impaired in indole-3-acetic acid production, indicating that hormone-related effects depend on an intact bacterial metabolic state [23].

### 3.5. Broader Functional Relevance of G. diazotrophicus Beyond Early Growth Promotion

Although sugarcane is the central biological system for the present manuscript, the functional behavior of *G. diazotrophicus* has also been documented in other plant contexts. Natural associations with wetland rice have been reported, indicating that the ecological competence of the bacterium is not absolutely restricted to sugarcane [24]. Likewise, antagonistic activity against *Xanthomonas albilineans* and the induction of defense-related responses in sugarcane have been described, suggesting that the bacterium can influence host performance through additional interaction channels beyond early growth promotion alone [25,26]. Moreover, *G. diazotrophicus* has been shown to solubilize zinc compounds, which adds a micronutrient-related dimension to its functional profile [27].

These studies are relevant to the present Discussion because they help contextualize the current results within a broader functional landscape. The early seedling responses described here are therefore compatible with a bacterium that combines diazotrophic capacity, developmental modulation, antagonistic potential, and nutrient-related effects. However, this broader literature does not diminish the specificity of the sugarcane system; rather, it reinforces the interpretation that the observed response architecture likely arises from the convergence of several bacterial functions operating simultaneously in a host-dependent manner [24,25,26,27].

### 3.6. Treatment Structure Matters, but Greater Structural Complexity Does Not Necessarily Translate into Stronger Plant Performance

A major result of the present study is that treatment structure did not behave as a uniformly positive determinant of final plant outcome. In the treatment contrasts, multivariate fingerprint, and semi-mechanistic model, greater inoculum structural complexity was not associated with stronger final biomass. The negative conditional contribution of the structure core indicates that increasing the number of isolate components did not produce a proportional improvement in early seedling performance. This finding is biologically plausible when interpreted through the literature on colonization and persistence.

Reporter-gene studies have shown that *G. diazotrophicus* colonization is spatially dynamic and tissue-dependent, indicating that effective establishment in the host is not simply a matter of bacterial presence, but of successful persistence and localization [28]. In parallel, exopolysaccharide production has been shown to be essential for biofilm formation and plant colonization, demonstrating that the capacity to remain functionally associated with the host depends on more than the inoculum dose itself [29]. Together, these studies suggest that increasing structural complexity may fail to improve plant performance if that complexity does not enhance colonization efficiency or if it imposes internal constraints on the conversion of microbial presence into host benefit.

This interpretation is further supported by work showing that phosphate availability enhances levan production in *G. diazotrophicus*, highlighting the role of extracellular carbohydrate metabolism in persistence-related traits [30]. In addition, transcriptomic evidence under drought conditions indicates that association with *G. diazotrophicus* can alter hormone-related pathways and stress responses in the host, reinforcing the view that plant outcome depends on the quality of the interaction rather than on inoculation structure alone [31]. Thus, the negative structure-core coefficient should be interpreted as evidence that consortium size alone was not a reliable predictor of early biomass response.

### 3.7. Comparison with Previous Sugarcane Studies Supports the Interpretation of a Treatment-Dependent and Context-Sensitive Response

The current data are broadly consistent with the view that the sugarcane–*G. diazotrophicus* association is real but conditional. Previous work showed that nitrogen fertilization can reduce bacterial populations through plant-mediated physiological effects [32], and later studies identified genes directly involved in indole-3-acetic acid biosynthesis in strain Pal5, thereby strengthening the mechanistic basis for treatment-dependent developmental effects [33]. These findings are fully compatible with the present results, where treatment identity explained a substantial proportion of the multivariate variation, but not all treatments translated stronger upstream signals into the same phenotypic outcome.

The same logic applies to stress adaptation and persistence. In *G. diazotrophicus*, resistance to abiotic stress has been associated with extracytoplasmic responses involving nutrient uptake, multidrug efflux systems, oxidative-stress response, and protein-quality-control mechanisms under cadmium exposure, indicating that persistence-related physiology may shape how effectively the bacterium converts its functional repertoire into plant-associated performance [34]. This supports the view that inoculum validation and exposure intensity are necessary descriptors, but not sufficient predictors of plant performance.

More applied studies also support this interpretation. Inoculation of sugarcane with endophytic diazotrophic bacteria has been associated with changes in yield, biological nitrogen fixation, and industrial characteristics [35], while endophytic bacterial inoculation has also been shown to improve nutrient-use efficiency and modulate foliar nitrogen-related metabolites in sugarcane seedlings [36]. Taken together, these studies reinforce the idea that plant benefit is usually expressed as a context-dependent integration of several growth-related processes rather than as a universal increase in one single trait [32,33,34,35,36].

### 3.8. The Multivariate and Semi-Mechanistic Framework Increases Interpretive Resolution and Defines the Proper Scope of the Manuscript

The semi-mechanistic model should be interpreted as an explanatory framework rather than as a complete causal reconstruction. Its value lies in showing that final biomass emerges from the interaction among validation, exposure, structure, and phenotype, and not from any one of these dimensions in isolation. This interpretation gains additional support from recent evidence showing that a functional *nifD* gene is required for optimal plant growth promotion by *G. diazotrophicus* in tomato, confirming that specific bacterial functions remain central to the expression of plant benefit even within a broader multifunctional framework [37].

Several limitations should nevertheless be stated explicitly. First, this study focuses on early seedling performance under greenhouse conditions, so the conclusions should be interpreted as evidence of early vigor architecture rather than as proof of field-scale agronomic superiority. Second, although the manuscript is informed by the known functional biology of *G. diazotrophicus*, the present dataset does not directly quantify *in planta* nitrogen fixation flux, hormone concentration, bacterial tissue density, or transcriptomic responses. Third, the semi-mechanistic model remains intentionally conservative: it captures the major explanatory structure of the dataset, but does not claim to resolve every mechanistic branch operating in the interaction.

These limitations also define the manuscript’s most useful application. The present study should be understood as a mechanistically informed greenhouse benchmark for early sugarcane seedling responses to *G. diazotrophicus*. It provides a robust basis for selecting biologically promising treatment profiles, for identifying which components of growth are most responsive at early stages, and for guiding the next generation of work toward direct colonization assays, isotope-based nitrogen fixation measurements, targeted phytohormone profiling, microscopy-based localization, and field validation. In this sense, the study contributes not only biological evidence, but also an analytical framework that can be transferred to future plant–microbe interaction studies.

## 4. Materials and Methods

### 4.1. Experimental Design and Treatment Structure

A greenhouse experiment was conducted to evaluate early sugarcane seedling responses to *G. diazotrophicus* under a treatment-structured inoculation framework. The analytical dataset comprised 350 observations distributed across eight treatments (T1–T8), including seven inoculated configurations and one non-inoculated control, with a near-balanced design of 43–44 observations per treatment. All plants were evaluated at 30 days after inoculation, and all inoculated treatments were applied using the same inoculation route, namely immersion inoculation for 1 h, under a plastic-covered greenhouse system and a shared soil substrate condition. Seedling phenotyping followed a trait-oriented logic focused on early morphological performance, biomass integration, and allocation, consistent with standardized plant trait measurement principles and biomass partitioning concepts [38,39]. Sugarcane seedlings derived from true seed (sexual seed) were obtained from the experimental propagation workflow and selected at a uniform early developmental stage before inoculation. Selection criteria included healthy appearance, absence of mechanical damage or visible contamination, homogeneous vigor, and comparable initial shoot and root development. Before treatment allocation, plant material was acclimated under phase-specific conditions: sprouting/photo-thermotherapy at 35–40 °C for 20 days, followed by greenhouse and tray-based vegetative development at 25–32 °C. Relative humidity was maintained at 80–90% during initial hardening and gradually reduced to 50–60% during final tray adaptation. Irrigation was applied two to three times per day as fine watering to maintain substrate moisture without saturation, while tray drainage was ensured to avoid root asphyxia and fungal proliferation. All treatments were grown in the same mineral substrate, coded as s-F1, composed of washed river sand, exfoliated vermiculite, and horticultural perlite at a 2:1:1 ratio (*v*/*v*/*v*). Each experimental unit contained 1000 mL of substrate, consisting of 500 mL of double-washed river sand, 250 mL of vermiculite, and 250 mL of perlite. This formulation was selected to provide adequate drainage, macroscopic porosity, and root aeration while minimizing external nutrient interference during the early seedling assay. The s-F1 substrate was used without organic amendments or nitrogen fertilizer in order to reduce the input of exogenous combined nitrogen during inoculum evaluation. Its mineral composition was mainly associated with silica-rich sand, aluminosilicate vermiculite, and expanded perlite. Therefore, the substrate was considered a low-nitrogen, low-organic-input matrix suitable for evaluating early sugarcane seedling responses under controlled greenhouse conditions. All treatments were maintained under the same substrate and management regime. The treatment structure is summarized in Table 2.

The treatment structure was designed to compare seven inoculated formulations against one non-inoculated control while preserving the same inoculation route, substrate, greenhouse management, and evaluation window across treatments. The inoculated treatments differed in the number and combination of isolate components, thereby generating a controlled gradient of inoculum structural complexity. This structure allowed the experiment to evaluate whether early sugarcane seedling responses were associated only with inoculation presence or also with the architecture of the inoculum formulation. The non-inoculated distilled-water treatment, T8, was used as the operational control for baseline greenhouse performance under the same substrate and management conditions.

### 4.2. Bacterial Material, Inoculum Recovery, and Operational Preservation

The bacterial material corresponded to CINCAE-preserved sugarcane-associated isolates previously assigned to *G. diazotrophicus* through institutional sequence-based taxonomic analysis. The CINCAE sequencing report was treated as the primary taxonomic support for maintaining the species-level assignment used in this manuscript. Therefore, the present study did not attempt to reclassify the isolates taxonomically. Instead, the experimental workflow focused on recovering, preserving, standardizing, and validating the biological material before greenhouse application. The operational aim of this stage was to ensure that the inoculum used for seedling exposure remained viable, traceable, and experimentally stable throughout the pre-inoculation workflow. Culture recovery, subculture handling, and aliquot preservation were performed under aseptic conditions in a laminar-flow workspace. Incubation of recovery media was carried out in a BINDER BD 56 standard incubator (BINDER GmbH, Tuttlingen, Germany), selected for its high temperature stability and routine incubation reliability. Mixing and short-term homogenization of culture suspensions were performed using a JOANLAB Vortex Mixer VM-400Max (JOANLAB Equipment, Huzhou, China), whereas small-volume centrifugation steps were conducted in a JOANLAB Mini Centrifuge MC-12K (JOANLAB Equipment, Huzhou, China). Preserved inoculum aliquots were maintained in sterile microtubes under refrigerated storage until standardization and application.

Operational preservation and pre-inoculation handling were documented photographically and incorporated into the biological validation sequence shown in Figure 2. This stage was designed to preserve continuity between inoculum recovery, preservation, and greenhouse application, thereby ensuring that the biological material evaluated in seedlings corresponded to a recoverable and experimentally controlled inoculum rather than to an undefined microbial input. The general logic of controlled inoculation and plant association follows the established experimental framework used for sugarcane–*G. diazotrophicus* systems [40,41].

### 4.3. Phenotypic and Molecular Validation of the Inoculum

Before plant inoculation, the inoculum was validated through a combined phenotypic and molecular workflow. The phenotypic validation included positive recovery in LGI-P semisolid medium, LGI-P solid medium, and PDA, together with a Gram-negative reaction, a biochemical profile compatible with the target inoculum, and phenotypic and molecular features compatible with the target *diazotrophic* inoculum, without treating the PCR profiles as direct evidence of nifH amplification or nitrogenase activity. These outputs were encoded as binary variables (0/1) in the analytical matrix and were later integrated into the validation core used in the multivariate and semi-mechanistic stages.

Gram staining and routine microscopic inspection were performed using a ZEISS Primostar 3 upright microscope (Carl Zeiss Microscopy GmbH, Jena, Germany). Molecular validation was based on three supportive PCR profiles corresponding to expected amplicon sizes of 218 bp, 1491 bp, and 395 bp. PCR amplification was carried out using a Mastercycler X50s thermocycler (Eppendorf SE, Hamburg, Germany). DNA concentration and optical reading support for inoculum standardization were obtained with a UV/Vis spectrophotometer (JOANLAB Equipment, Huzhou, China). Electrophoretic separation was performed in a horizontal gel electrophoresis chamber connected to a standard laboratory power supply, and gel visualization was conducted with a GelDoc Go Gel Imaging System (Bio-Rad Laboratories, Hercules, CA, USA).

The target region, primer sequences, expected amplicon size, marker specificity, and analytical purpose of each PCR assay are summarized in Table 3.

The 16S rRNA gene region was treated as a foundational bacterial identity marker following standard phylogenetic amplification logic [44,45], whereas the 218 bp 16S–23S rDNA intergenic spacer/ITS amplification was used as the species-associated marker-level support for the CINCAE-confirmed *G. diazotrophicus* isolate [46]. The validation logic was further supported by the previously documented genetic structure of *G. diazotrophicus* populations and strain-level variation [47,48]. The resulting electrophoretic fingerprints are presented in Figure 3. PCR validation was used as an operational molecular confirmation step before plant inoculation. Three reproducible amplicon profiles were considered: 218 bp, approximately 1491 bp, and 395 bp. The approximately 1491 bp and 395 bp products were interpreted as broad bacterial 16S rRNA gene identity markers, whereas the 218 bp product provided the most specific *G. diazotrophicus*-associated 16S–23S rDNA intergenic marker support within the inoculum validation workflow. Negative extraction/control lanes were included to rule out obvious contamination during the PCR workflow. The lane labelled Cp in the original gel layout did not show a clearly visible positive-control band in the displayed image and was therefore not used as interpretive evidence. Consequently, PCR-based support was limited to the reproducible amplicon profiles observed in the sample lanes and interpreted together with the CINCAE sequencing report and culture recovery evidence.

### 4.4. Inoculum Standardization and Greenhouse Inoculation

Before plant application, each inoculum suspension was homogenized and standardized by optical density at 600 nm (OD600) using a UV/Vis spectrophotometer (JOANLAB Equipment, Huzhou, China). The standardized inoculum corresponded to OD600 = 0.82 ± 0.04, equivalent to 1.35 × 10^8^ ± 0.21 × 10^8^ CFU mL^−1^ based on viable-cell calibration. CFU mL^−1^ was used as the operational viable-density standard before plant application, whereas OD600 was retained as the analytical exposure descriptor in the multivariate dataset. Seedlings assigned to T1–T7 were immersed in the corresponding inoculum suspension for 1 h. Seedlings assigned to T8 were immersed in sterile distilled water for the same duration. After immersion, all seedlings were transferred to the common s-F1 substrate and maintained under the same greenhouse regime until the final phenotypic assessment at 30 days after inoculation.

The greenhouse phase was conducted under a plastic-covered greenhouse system and a common substrate condition coded in the dataset as s-F1. The evaluation window was intentionally restricted to the early seedling stage in order to capture coordinated changes in elongation, biomass integration, and allocation before mature agronomic traits could confound the response. This timing is consistent with early plant development studies centered on trait integration and biomass partitioning [38,39].

### 4.5. Phenotypic Measurements and Derived Variables

The phenotypic design included baseline and final measurements of shoot length and root length, together with terminal shoot mass, root mass, total biomass, and root-to-shoot ratio. Baseline measurements were recorded immediately before or at the start of the greenhouse phase, while final measurements were obtained at day 30. Linear measurements were taken with a digital caliper, model 500-196-30 (Mitutoyo, Kawasaki, Japan), and terminal biomass was measured with a Sartorius Entris II BCE224I-1S analytical balance (Sartorius Lab Instruments GmbH & Co. KG, Göttingen, Germany), with 0.1 mg readability and internal calibration.

The following derived variables were calculated from the raw observations:

(1) $${\mathrm{Shoot}\;\mathrm{Gain}}_{i}={\mathrm{Final}\;\mathrm{Shoot}}_{i}-{\mathrm{Baseline}\;\mathrm{Shoot}}_{i}$$

(2) $${\mathrm{Root}\;\mathrm{Gain}}_{i}={\mathrm{Final}\;\mathrm{Root}}_{i}-{\mathrm{Baseline}\;\mathrm{Root}}_{i}$$

(3) $${\mathrm{Total}\;\mathrm{Biomass}}_{i}={\mathrm{Shoot}\;\mathrm{Weight}}_{i}+{\mathrm{Root}\;\mathrm{Weight}}_{i}$$

(4) $${\mathrm{Root}:\mathrm{Shoot}\;\mathrm{Ratio}}_{i}=\frac{{\mathrm{Root}\;\mathrm{Weight}}_{i}}{{\mathrm{Shoot}\;\mathrm{Weight}}_{i}}$$

These equations follow standard trait-based and biomass allocation logic for early plant performance analysis [38,39]. Because the study focused on early vigor rather than terminal yield, gain variables were prioritized whenever the objective was to isolate growth occurring during the evaluation interval rather than to rely on endpoint size alone.

### 4.6. Analytical Engineering of Validation, Exposure, Structure, Phenotypic, and Outcome Cores

To interpret the experiment as an integrated biological system, the observed variables were organized into analytical cores before multivariate and model-based analysis. The validation core represented biological traceability of the inoculum and included identity validation and molecular validation variables. The exposure domain included OD600, colonization potential, and dose plausibility for multivariate fingerprinting. For the semi-mechanistic biomass model, the exposure core was conservatively restricted to OD600 and dose plausibility, because colonization potential was a treatment-derived descriptor partially redundant with upstream inoculum structure. The structure core was represented by strain count, which captured inoculum compositional complexity. The phenotypic core included shoot gain, root gain, and root-to-shoot ratio, whereas the outcome core was anchored by total biomass. Continuous variables were standardized as z-scores before integration:

(5) $$z_{ij}=\frac{x_{ij}-{\overset{¯}{x}}_{j}}{{SD}_{j}}$$

where *xᵢⱼ* is the value of variable *j* in observation *i*, ${\overset{¯}{x}}_{j}$ is the mean of variable *j*, and *SDⱼ* is the standard deviation of variable *j*. Treatment-level standardized fingerprints were calculated from mean z-scores by treatment. For the core-level contribution profile shown in Figure 6C, absolute standardized treatment means were normalized within each treatment so that the relative contribution of each analytical core could be compared without collapsing the dataset into a single undifferentiated score. This output should be interpreted as a standardized analytical signal profile, not as microbial abundance.

### 4.7. Treatment-Level Contrast Analysis

Treatment-level contrasts were performed to separate absolute endpoint performance from inoculated-treatment ranking. First, each inoculated treatment (T1–T7) was compared against the non-inoculated water control (T8) for total biomass, shoot gain, and root gain. This contrast addressed whether any inoculated treatment reached or exceeded the strongest absolute endpoint control. Second, inoculated treatments were ranked internally by comparing total biomass against T2, which was used as the best-performing inoculated reference treatment based on the phenotypic and multivariate results.

For each contrast, mean differences were calculated together with bootstrap 95% confidence intervals, following a resampling-based approach for estimating uncertainty around treatment effects [49]. Pairwise statistical support was assessed using Welch-type comparisons, which provide a more robust alternative to equal-variance comparisons when homogeneity of variance cannot be assumed [50]. To reduce type I error inflation across multiple pairwise contrasts, *p*-values were adjusted using the Holm step-down procedure within the family of treatment comparisons [51]. These outputs are presented in Figure 5.

### 4.8. Multivariate Profiling, Clustering, and PERMANOVA

The multivariate fingerprint was constructed to evaluate whether the treatments differed as integrated response profiles rather than as isolated single-variable outcomes. To address this objective, a reduced analytical matrix was assembled using non-redundant variables representing exposure, inoculation structure, plant phenotype, and final outcome. The matrix included OD600, Strain Count, Colonization Potential Index, Dose Plausibility Score, Shoot Gain (mm), Root Gain (mm), Total Biomass (mg), and Root Shoot Ratio. Identity validation and molecular validation variables were not included in this PCA–PERMANOVA matrix because they were binary inoculum-verification descriptors rather than continuous treatment-response variables; these variables were used to support inoculum traceability and PCR-based validation, but not to drive multivariate treatment separation.

Before ordination and distance-based testing, all variables were standardized as z-scores to place measurements with different units and scales on a comparable analytical basis [52]. Principal component analysis (PCA) was then applied to the standardized matrix to visualize the main axes of treatment separation and the direction of the contributing variable vectors in reduced-dimensional space [53,54]. The PCA biplot shown in Figure 6A was used as an exploratory ordination tool to determine whether treatments occupied distinct regions of the multivariate response space.

K-means clustering was applied to the PCA-transformed scores using the Hartigan–Wong algorithm [55]. The clustering step was used to support visualization of treatment grouping patterns in the ordination space, not as a replacement for formal hypothesis testing. Internal partition behavior was inspected using silhouette-based logic to verify whether the selected grouping structure was coherent with the distribution of observations in the reduced multivariate space [56].

To complement the ordination, treatment-level standardized fingerprints were generated as a z-score heatmap across the selected variables. This output allowed direct inspection of which variables contributed most strongly to each treatment profile, especially for distinguishing treatments with higher exposure or structure-related signals from those with stronger shoot, root, biomass, or allocation responses. In addition, standardized variables were aggregated into four analytical cores: exposure, structure, phenotype, and outcome. These core-level summaries were used to produce the relative contribution panel in Figure 6C.

The global treatment effect on multivariate structure was evaluated using PERMANOVA with 999 permutations [57]. Euclidean distance was used because the analysis was performed on a standardized continuous matrix containing phenotypic, exposure, structure, and outcome variables rather than compositional abundance data. To avoid interpreting treatment separation without checking the dispersion assumption, treatment-wise distances to group centroids were calculated and visualized as a multivariate dispersion diagnostic following distance-based homogeneity-of-dispersion logic [58]. Interpretation of PERMANOVA considered the known limitation that location effects and dispersion effects may be partially confounded in heterogeneous multivariate systems [59]. The corresponding ordination, treatment fingerprints, core contribution summaries, and dispersion diagnostics are shown in Figure 6.

### 4.9. Semi-Mechanistic Biomass Modelling and Diagnostic Assessment

A semi-mechanistic ordinary least squares model was fitted to explain total biomass from four standardized analytical cores: validation, exposure, inoculum structure, and phenotypic performance. The model was designed as an explanatory framework, not as direct mechanistic proof of nitrogen fixation, auxin production, or *in planta* colonization density. Instead, it was used to evaluate whether final biomass could be statistically explained by experimentally traceable components of the inoculation workflow, including inoculum validation, exposure intensity, treatment structure, and early seedling growth response. Total biomass was used as the response variable and was z-standardized before coefficient estimation. All predictor variables entering the analytical cores were also z-standardized to place variables measured on different scales on a common analytical basis. The final model was specified as:

(6) $$zBiomass_{i}=\beta_{0}+\beta_{1}Validation_{i}+\beta_{2}Exposure_{i}+\beta_{3}Structure_{i}+\beta_{4}Phenotypic_{i}+\varepsilon_{i}$$

where $zBiomass_{i}$ represents the z-standardized total biomass of observation i, $Validation_{i}$ represents inoculum identity and molecular support, $Exposure_{i}$ represents inoculum exposure intensity, $Structure_{i}$ represents inoculum compositional complexity, and $Phenotypic_{i}$ represents integrated early seedling growth response. The validation core was calculated as:

(7) $$Validation_{i}=\frac{z(Identity\;validation_{i})+z(Molecular\;validation_{i})}{2}$$

Identity validation represented the taxonomic assignment of the CINCAE-preserved *G. diazotrophicus* material used in the assay, whereas molecular validation represented the supportive PCR-based confirmation workflow applied before greenhouse inoculation. The validation core was therefore used to represent biological traceability of the inoculum, not as an independent claim of functional activity. The exposure core was calculated as:

(8) $$Exposure_{i}=\frac{z(OD600_{i})+z(Dose\;plausibility_{i})}{2}$$

OD600 was retained as the treatment-level analytical exposure descriptor, while dose plausibility represented the consistency of the operational inoculum application scheme. Before greenhouse application, the inoculum suspensions were additionally supported by viable-cell standardization, corresponding to 1.35 × 10^8^ ± 0.21 × 10^8^ CFU mL^−1^. This CFU estimate was used to confirm viable inoculum density before plant application. However, CFU mL^−1^ was not included as an independent predictor in the model because it functioned as a standardization control rather than as an observation-level response variable in the analytical matrix. The structure core was calculated as:

(9) $$Structure_{i}=z\left(Strain\;count_{i}\right)$$

Strain count was used to represent inoculum compositional complexity across treatments. This variable allowed the model to test whether treatments with a higher number of isolate components necessarily produced higher biomass. The phenotypic core was calculated as:

(10) $$Phenotypic_{i}=\frac{z(Shoot\;gain_{i})+z(Root\;gain_{i})+z(Root\text{-}to\text{-}shoot\;ratio_{i})}{3}$$

This core integrated early growth and allocation descriptors into a single standardized plant-performance variable. Shoot gain and root gain represented absolute elongation responses from baseline to day 30, whereas the root-to-shoot ratio represented allocation balance. In the multivariate PCA–PERMANOVA fingerprint shown in Figure 6, validation descriptors were excluded because that analysis focused on response separation among treatments. In contrast, the semi-mechanistic model shown in Figure 8 retained the Validation core as a traceability-control predictor to evaluate whether inoculum validation contributed explanatory structure after accounting for exposure, strain structure, and phenotypic response.

The model was fitted using ordinary least squares regression. Standardized coefficients, standard errors, 95% confidence intervals, and *p*-values were estimated, and HC3 robust standard errors were used to reduce sensitivity to heteroscedasticity. Model quality was evaluated using *R*^2^, adjusted *R*^2^, root mean square error, mean absolute error, observed-versus-predicted response, Q–Q residual diagnostics, residual distribution, residuals versus fitted values, variance inflation factors, leverage, and Cook’s distance.

Collinearity was assessed using variance inflation factors, with values below 5 considered acceptable for retaining predictors in the explanatory model. Influence diagnostics were examined using leverage and Cook’s distance to identify whether individual observations disproportionately affected the fitted model. The final model retained acceptable collinearity and was therefore interpreted as a semi-mechanistic explanatory summary of biomass response architecture across the evaluated treatments.

### 4.10. Model Diagnostics, Effect Size, and Collinearity Control

Model adequacy was assessed using complementary diagnostic procedures. Collinearity was examined using generalized variance inflation factor logic [60], with threshold interpretation informed by the cautionary treatment of common VIF rules of thumb [61]. Residual normality was inspected graphically and tested using the Shapiro–Wilk test [62]. Heteroscedasticity was assessed using the Breusch–Pagan test [63]. Influential observations were evaluated through Cook’s distance and leverage [64]. Local predictor effect size was summarized using Cohen’s $f^{2}$:

(11) $$f^{2}=\frac{R_{\mathrm{full}}^{2}-R_{\mathrm{reduced}}^{2}}{1-R_{\mathrm{full}}^{2}}$$

where $R_{\mathrm{full}}^{2}$ is the coefficient of determination of the full model and $R_{\mathrm{reduced}}^{2}$ is the coefficient of determination after removing the focal predictor [65]. When exploratory simultaneous inference was required for model-based contrasts or coefficient interpretation, general parametric-model procedures were followed [66]. Graphical diagnostics were used to complement scalar model summaries by revealing residual structure, leverage patterns, and influence behavior. The complete model diagnostic fingerprint is presented in Figure 8.

### 4.11. Software Environment and Reproducible Workflow

All data preprocessing, variable engineering, multivariate analysis, model fitting, and figure production were carried out in Python (v. 3.13.5) through a reproducible script-based workflow. Numerical array operations and matrix standardization were implemented with NumPy (v. 2.3.5) [67], while tabular data management and transformation were conducted with pandas (v. 2.2.3) [68]. Scientific routines associated with linear algebra, statistical preprocessing, and distance-based procedures were handled with SciPy (v. 1.17.0) [69]. Graphical outputs, including the phenotypic, multivariate, and diagnostic figures, were produced with Matplotlib (v. 3.10.8) [70]. The semi-mechanistic OLS model and associated diagnostic tests were implemented with statsmodels (v. 0.14.6) [71], and the correlation and core-level network structures were handled with NetworkX (v. 3.6.1) [72]. This Python-based workflow was used consistently across the analytical sequence represented in Figure 4, Figure 5, Figure 6, Figure 7 and Figure 8.

## 5. Conclusions

This study demonstrates that early sugarcane seedling responses to *G. diazotrophicus* inoculation should be interpreted as a treatment-dependent and multidimensional response rather than as a simple inoculated-versus-non-inoculated contrast. Under the evaluated greenhouse conditions, the non-inoculated water control T8 reached the highest absolute endpoint performance, particularly for shoot growth and total biomass. However, this result should not be interpreted as evidence against the biological relevance of *G. diazotrophicus*. Instead, it indicates that baseline seedling growth under the experimental conditions represented a strong absolute reference. Within the inoculated subset, T2 showed the most balanced response profile, combining favorable shoot gain, root gain, and total biomass, and therefore represented the most coherent inoculated treatment.

The integrated analytical framework used in this study helped clarify the relationship between inoculum validation, exposure intensity, treatment architecture, phenotypic expression, and biomass accumulation. The treatment contrasts, multivariate fingerprints, inoculated-treatment ranking, and semi-mechanistic biomass model consistently showed that stronger inoculum-related descriptors or greater isolate complexity did not necessarily translate into higher plant performance. In particular, the lower performance of structurally more complex treatments indicates that consortium size alone is not a reliable predictor of early seedling response. Instead, the results suggest that early growth performance depends on how effectively each inoculated configuration is translated into coordinated shoot, root, and biomass development.

At the biological level, these findings support a cautious interpretation of *G. diazotrophicus* as a growth-associated endophyte whose effects are context-dependent, trait-specific, and influenced by treatment structure. The study does not provide direct evidence of *in planta* nitrogen fixation, nitrogenase activity, bacterial tissue density, or phytohormone production; therefore, the proposed mechanisms should be understood as literature-supported interpretations rather than direct biochemical confirmation. Nevertheless, the results provide a useful greenhouse benchmark for identifying promising inoculated profiles and for refining future plant–endophyte assays.

Future studies should validate the most favorable inoculated configurations under expanded experimental conditions, including direct colonization assays, isotope-based nitrogen fixation measurements, targeted phytohormone profiling, microscopy-based localization, and field-scale evaluation. Overall, this work contributes both biological evidence and an analytical strategy for interpreting early sugarcane seedling responses to *G. diazotrophicus* through integrated phenotypic, multivariate, and semi-mechanistic approaches.

## Figures and Tables

**Figure 1:**
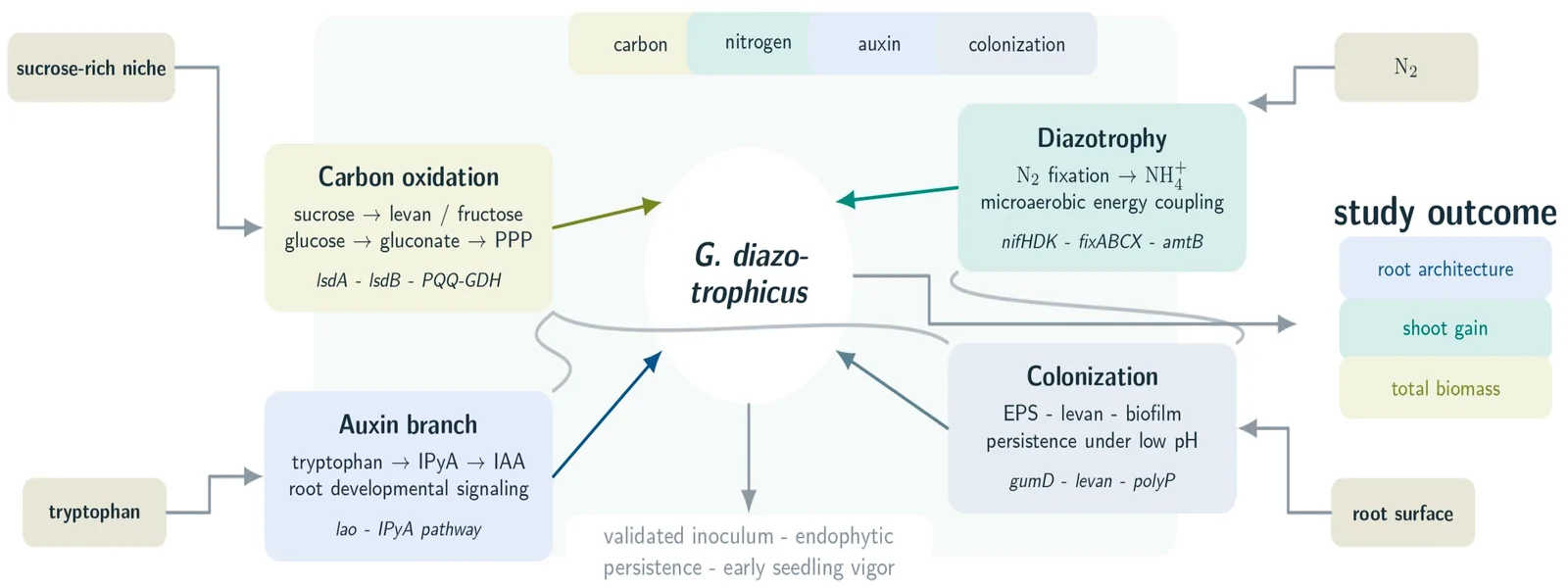
Metabolic-functional framework of *G. diazotrophicus* supporting the biological rationale of the present study. The scheme summarizes four interconnected functional branches relevant to early sugarcane seedling responses: carbon oxidation in a sucrose-rich niche, diazotrophy, tryptophan-dependent auxin-related signaling, and colonization/persistence mediated by extracellular polymers and biofilm-associated traits. The diagram is intended as a literature-grounded conceptual framework for interpreting the experimental response architecture evaluated in this work.

**Figure 2:**
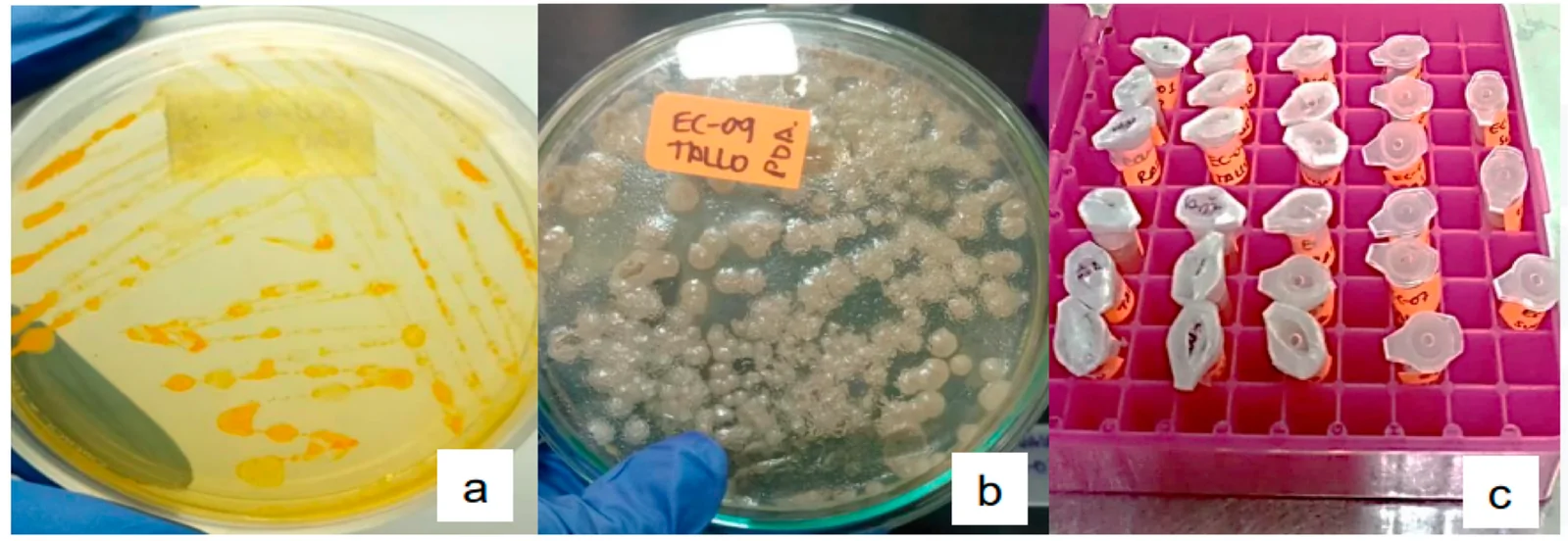
Culture morphology and preservation support of the validated *G. diazotrophicus* inoculum used in the experiment. (**a**) Representative colony morphology and growth recovery on solid medium. (**b**) Plate-level culture support showing active microbial growth prior to plant inoculation. (**c**) Preserved inoculum aliquots maintained under organized storage conditions for experimental standardization and downstream application. Together, these panels document the operational viability and traceability of the inoculum throughout the experimental workflow.

**Figure 3:**
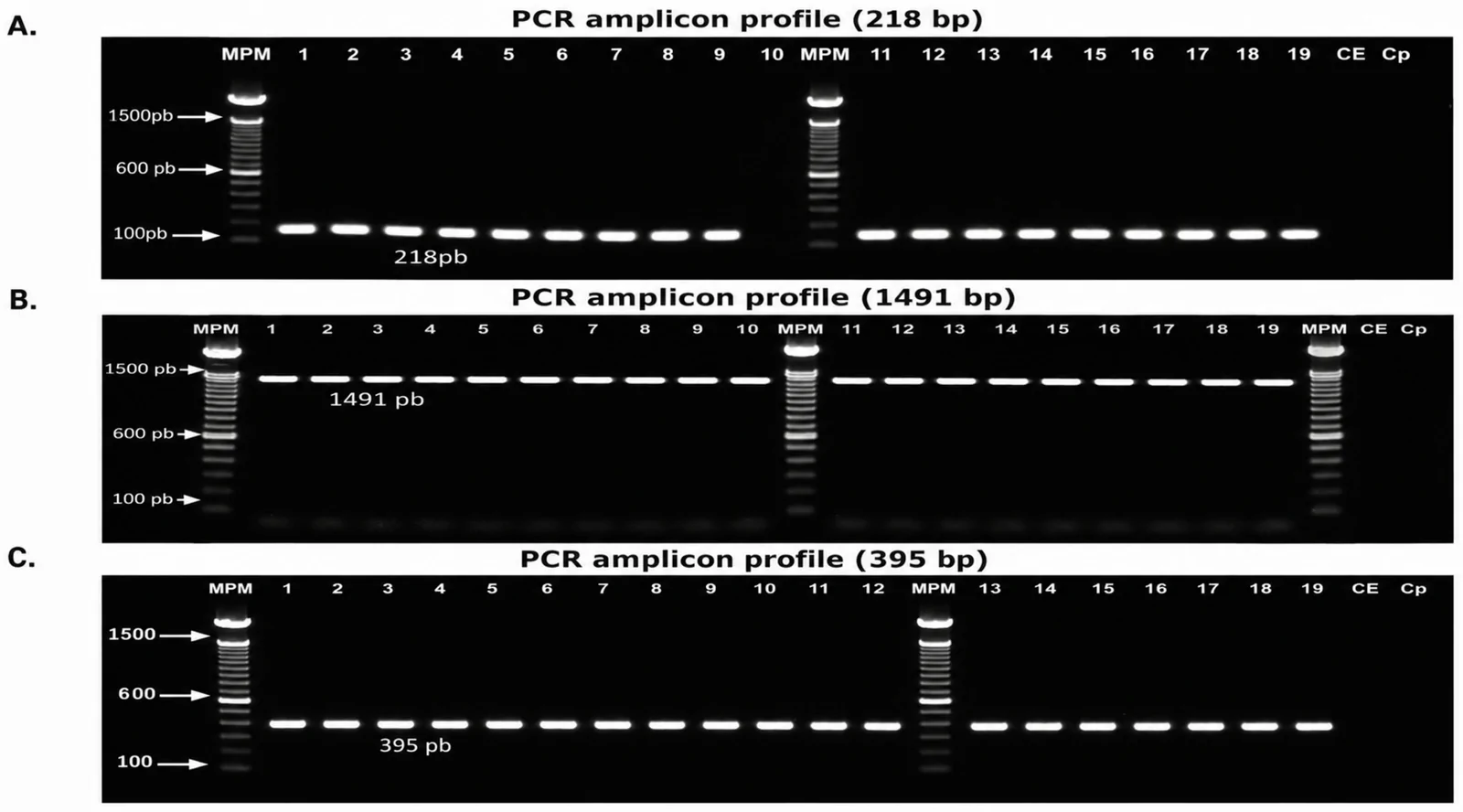
Consolidated PCR validation fingerprint of the *G. diazotrophicus* inoculum. (**A**) PCR amplicon profile corresponding to the 218 bp *G. diazotrophicus* 16S–23S rDNA internal transcribed spacer/intergenic spacer marker. (**B**) PCR amplicon profile corresponding to the approximately 1491 bp bacterial 16S rRNA near full-length fragment. (**C**) PCR amplicon profile corresponding to the 395 bp partial bacterial 16S rRNA fragment. MPM: molecular weight marker; CE: negative extraction control; Cp: control lane included in the original gel layout. Because no clear positive amplification band is visible in the displayed Cp lane, this lane was not used as interpretive evidence for inoculum validation.

**Figure 4:**
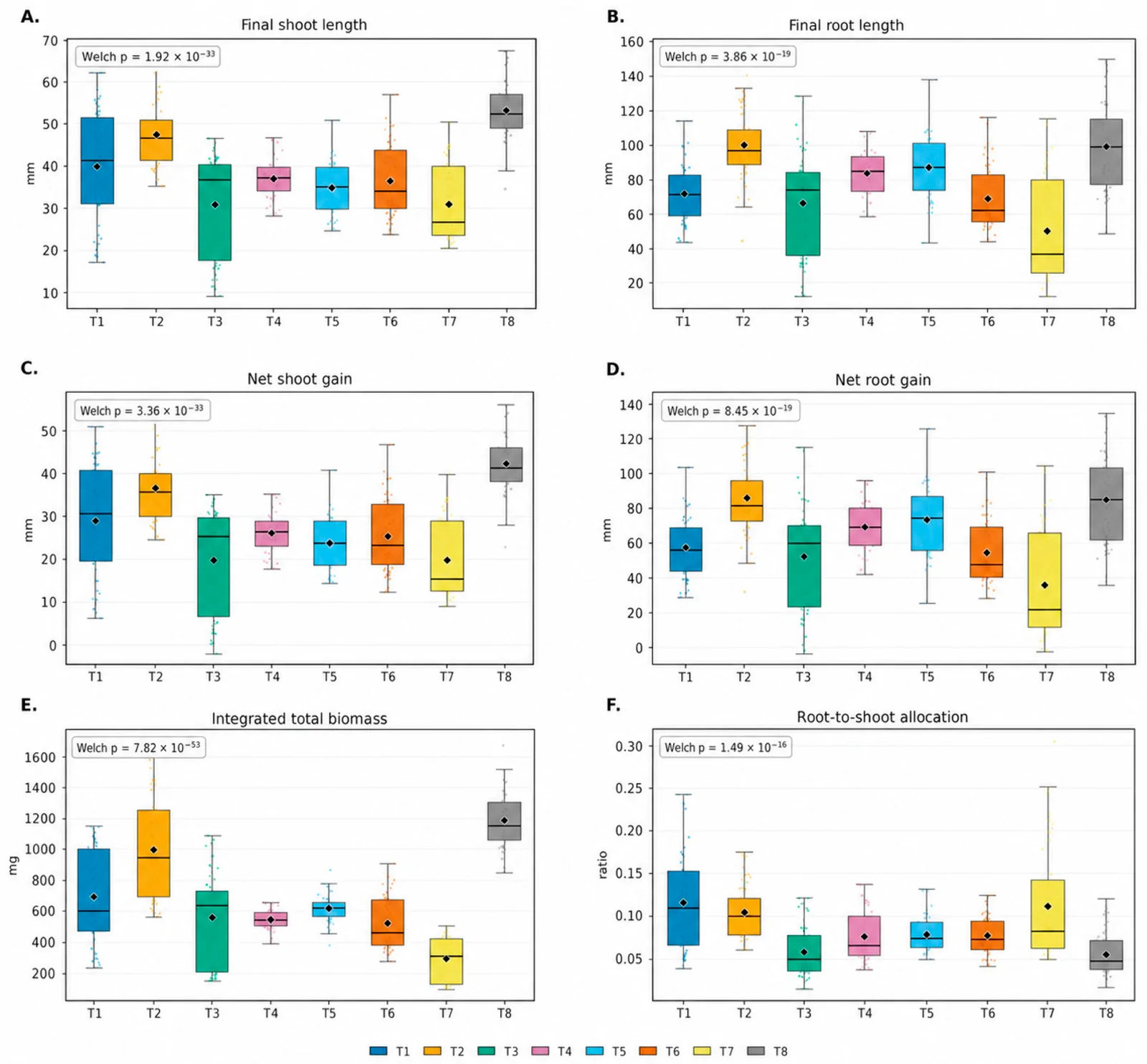
Treatment-dependent phenotypic response at day 30. (**A**) Final shoot length. (**B**) Final root length. (**C**) Net shoot gain. (**D**) Net root gain. (**E**) Integrated total biomass. (**F**) Root-to-shoot allocation. Boxplots show the distribution of individual observations by treatment; points represent individual seedlings; diamonds indicate treatment means. Welch’s ANOVA *p*-values are shown in each panel.

**Figure 5:**
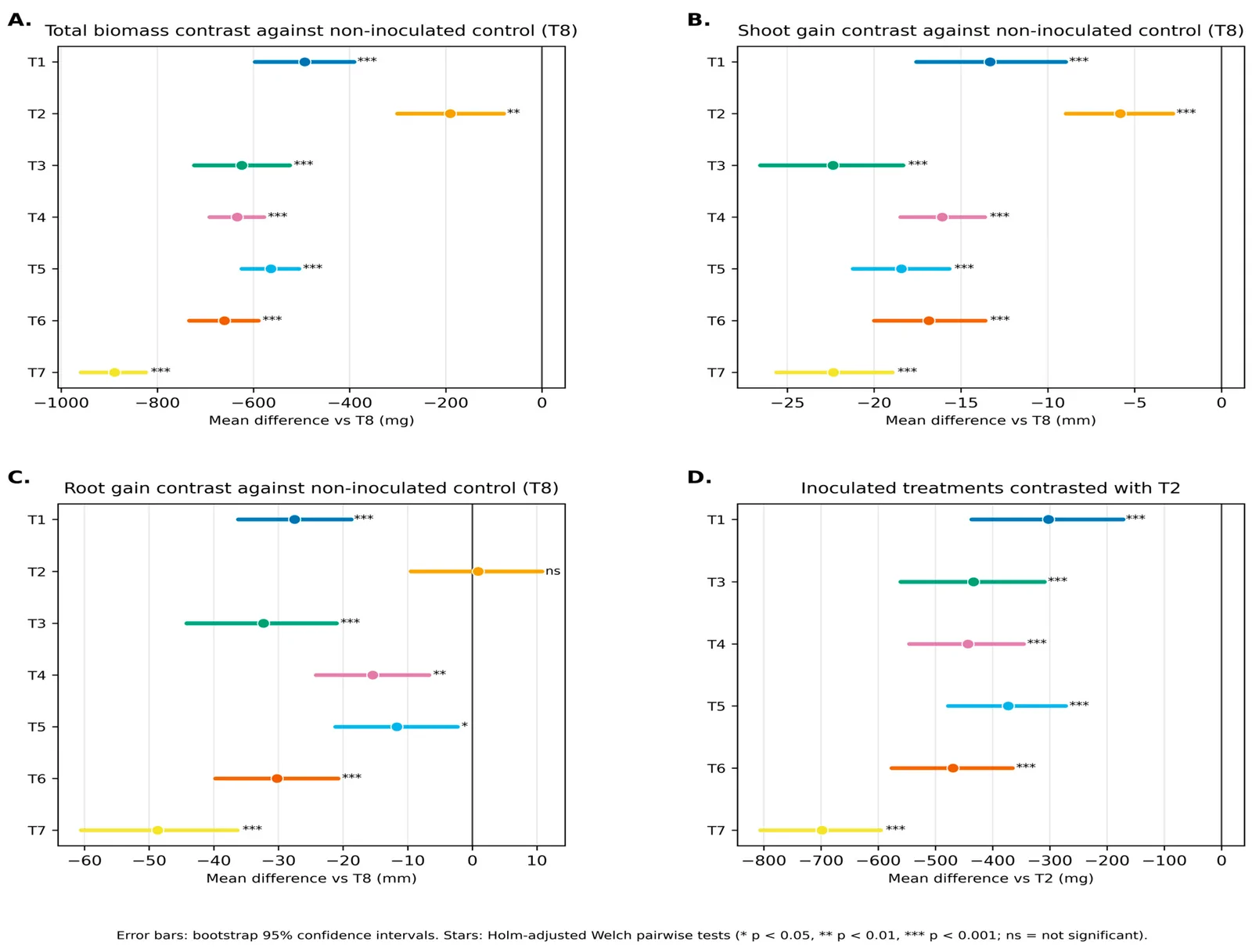
Treatment contrasts separating absolute control performance from inoculated-treatment ranking. (**A**) Mean total biomass differences between each inoculated treatment and the non-inoculated control T8. (**B**) Mean shoot gain differences between each inoculated treatment and T8. (**C**) Mean root gain differences between each inoculated treatment and T8. (**D**) Mean total biomass differences between the inoculated treatments and T2, used as the best-performing inoculated reference. Dots indicate mean differences; horizontal bars indicate bootstrap 95% confidence intervals. The vertical line at zero represents no difference from the reference treatment. Negative values indicate lower performance than the reference. Stars indicate Holm-adjusted Welch pairwise test significance: * *p* < 0.05, ** *p* < 0.01, *** *p* < 0.001; ns = not significant.

**Figure 6:**
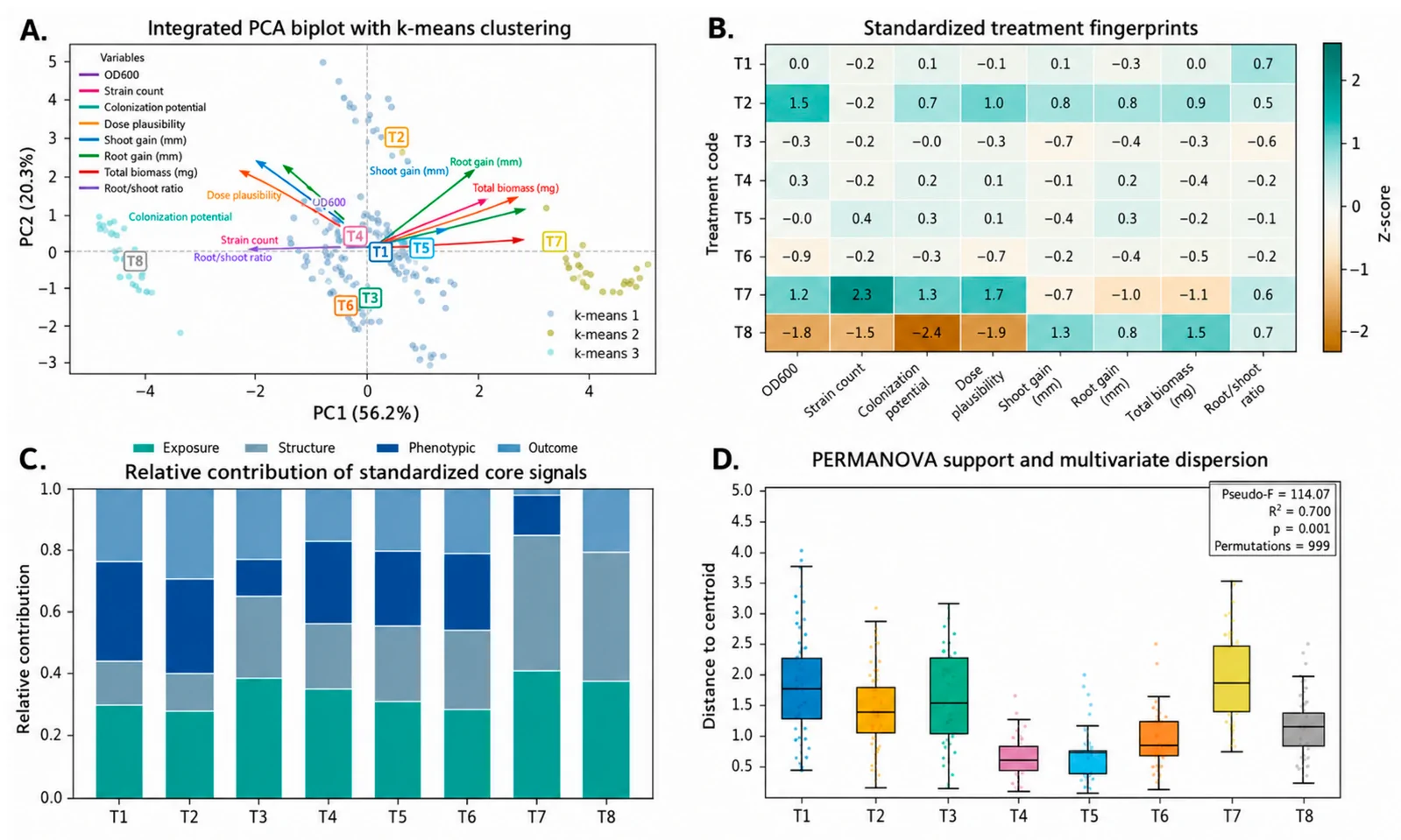
Multivariate fingerprint of the treatment-response matrix. (**A**) PCA biplot with k-means clustering and variable-vector direction. (**B**) Z-score heatmap of treatment fingerprints across OD600, strain count, colonization potential, dose plausibility, shoot gain, root gain, total biomass, and root-to-shoot ratio. (**C**) Relative contribution of standardized exposure, structure, phenotypic, and outcome cores. (**D**) PERMANOVA and multivariate dispersion diagnostics based on Euclidean distances from the standardized matrix.

**Figure 7:**
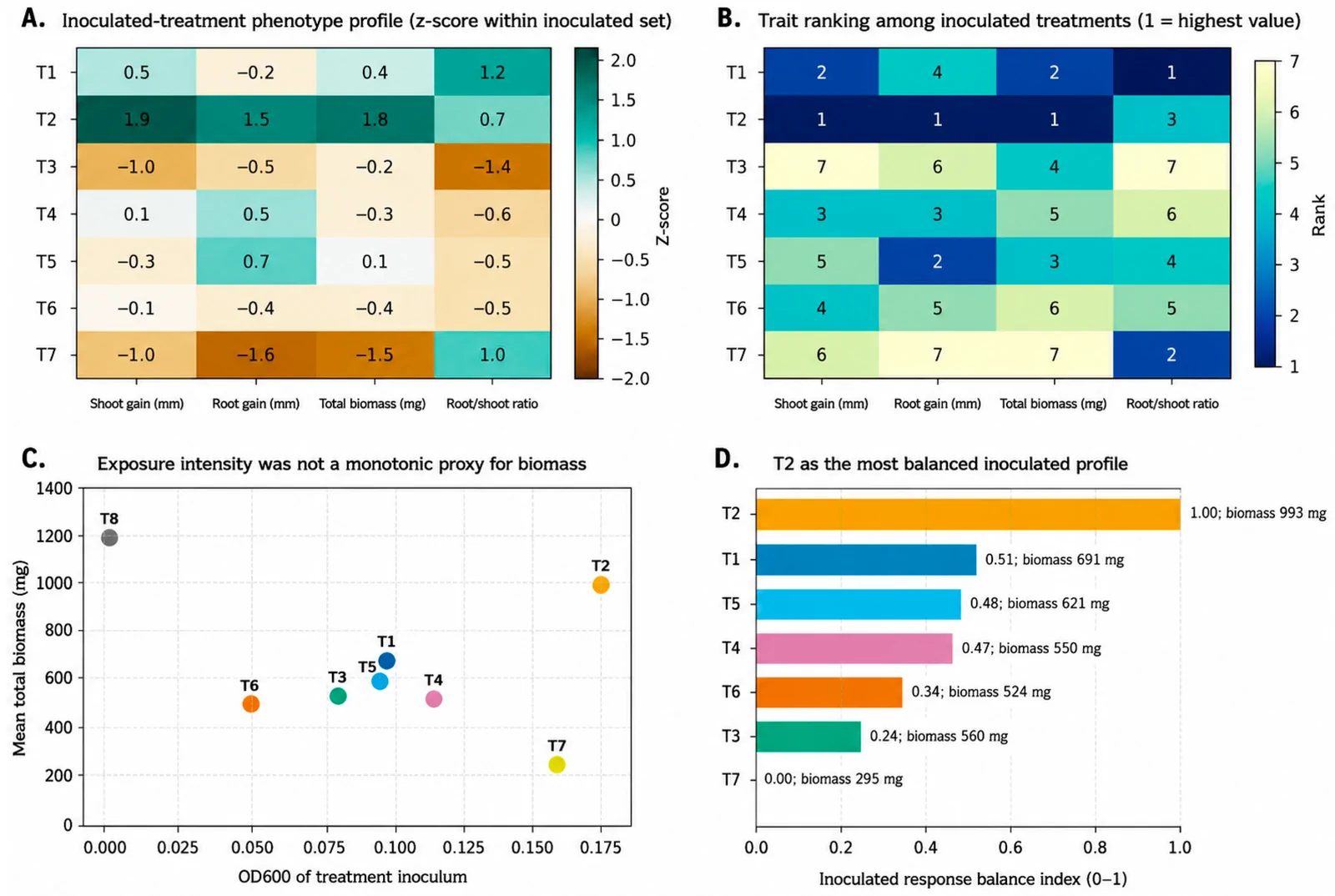
Inoculated-treatment ranking and exposure–performance decoupling. (**A**) Standardized phenotype profile of the inoculated treatments (T1–T7), expressed as z-scores within the inoculated subset for shoot gain, root gain, total biomass, and root-to-shoot ratio. (**B**) Trait ranking among inoculated treatments, where rank 1 indicates the highest value for each trait. (**C**) Relationship between inoculum OD600 and mean total biomass, showing that exposure intensity was not a monotonic proxy for biomass accumulation. T8 is included as the non-inoculated control reference. (**D**) Inoculated response balance index calculated from min–max normalized shoot gain, root gain, and total biomass within T1–T7. Higher values indicate a more balanced positive inoculated response across the main growth outputs.

**Figure 8:**
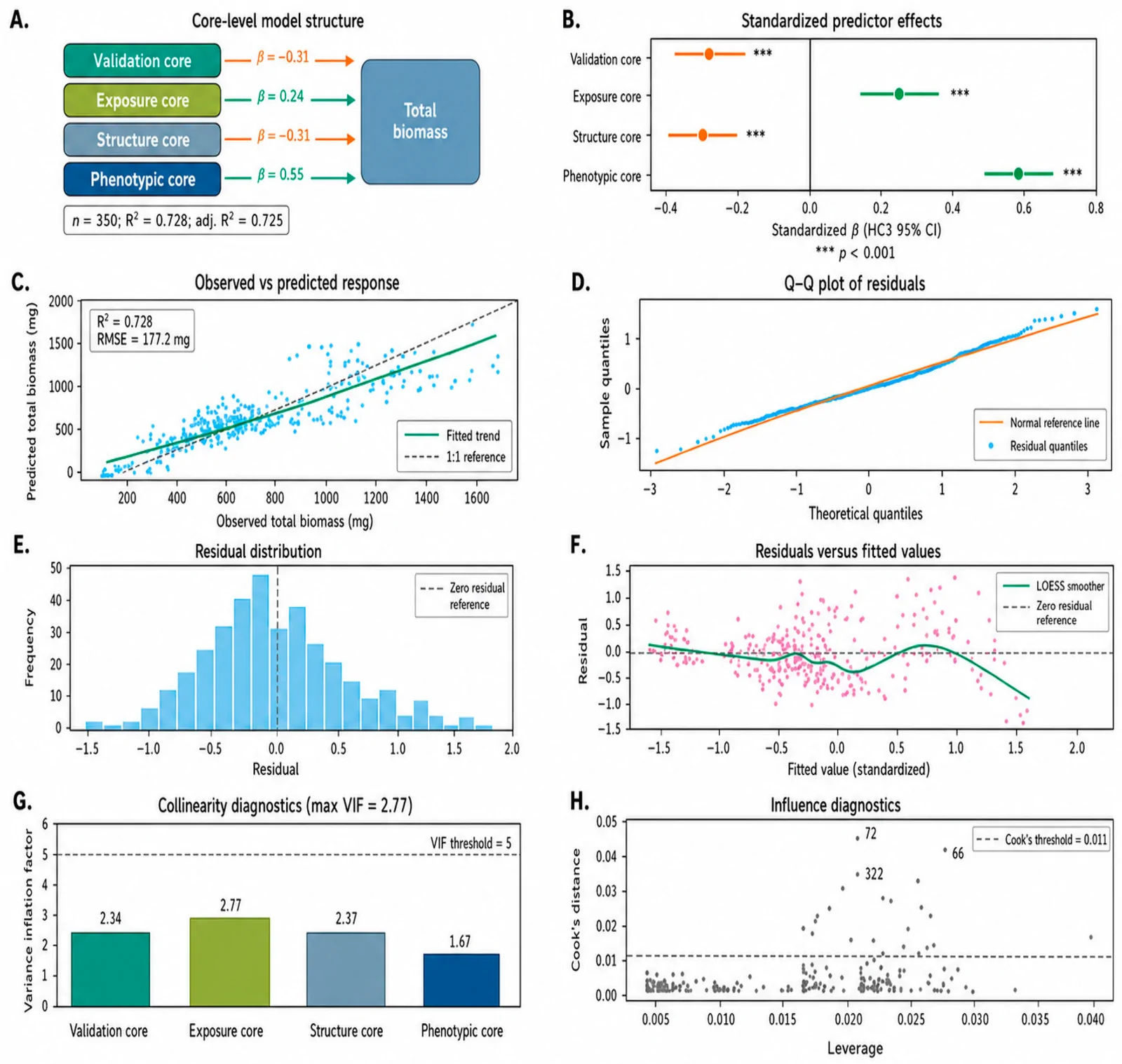
Semi-mechanistic biomass model and diagnostic structure. (**A**) Core-level model structure showing validation, exposure, inoculum structure, and phenotypic predictor cores explaining standardized total biomass. Validation was used only in this explanatory model as an inoculum-traceability core and was not included in the PCA–PERMANOVA response matrix shown in Figure 6. (**B**) Standardized predictor effects with 95% confidence intervals; *** indicates *p* < 0.001. (**C**) Observed versus predicted total biomass response; the green solid line represents the fitted trend, and the gray dashed line represents the 1:1 reference line. (**D**) Q–Q plot of residuals; the orange solid line represents the normal reference line. (**E**) Residual distribution; the gray dashed vertical line represents the zero-residual reference. (**F**) Residuals versus fitted values; the green solid line represents the LOESS smoother, and the gray dashed line represents the zero-residual reference. (**G**) Collinearity diagnostics based on variance inflation factors; the gray dashed horizontal line represents the VIF threshold of 5. (**H**) Influence diagnostics based on leverage and Cook’s distance; the gray dashed horizontal line represents Cook’s distance threshold.

**Table 1:** Standardized coefficients and collinearity diagnostics of the semi-mechanistic biomass model.

Predictor Core	Standardized β	Robust SE HC3	95% CI	z-Value	*p*-Value	VIF
Validation core	−0.307	0.044	−0.394 to −0.221	−6.963	3.33 × 10^−12^	2.34
Exposure core	0.239	0.048	0.145 to 0.333	4.998	5.79 × 10^−7^	2.77
Structure core	−0.310	0.038	−0.385 to −0.236	−8.175	2.95 × 10^−16^	2.37
Phenotypic core	0.546	0.035	0.478 to 0.613	15.783	4.05 × 10^−56^	1.67

Notes: Standardized β values were estimated from the core-level semi-mechanistic model shown in Figure 8. Robust standard errors were calculated using the HC3 estimator. The response variable was total biomass. The Validation core combined identity validation and molecular validation as inoculum-traceability descriptors; the Exposure core combined OD600 and dose plausibility; the Structure core was represented by strain count; and the Phenotypic core combined shoot gain, root gain, and root-to-shoot ratio. VIF = variance inflation factor.

**Table 2:** Treatment structure used in the greenhouse assay.

Treatment	Composition	Isolate Source	Operational Interpretation
T1	gt1 + gr1	Stem + root isolates	Two-component stem–root isolate formulation
T2	gt2 + gr2	Stem + root isolates	Two-component stem–root isolate formulation
T3	gt7 + gr7	Stem + root isolates	Two-component stem–root isolate formulation
T4	gt8 + gr8	Stem + root isolates	Two-component stem–root isolate formulation
T5	gt9 + gr9 + gh9	Stem + root + leaf isolates	Three-component stem–root–leaf isolate formulation
T6	gt85 + gr85	Stem + root isolates	Two-component stem–root isolate formulation
T7	gs1 + gs2 + gs7 + gs8 + gs9 + gs85-92	Rhizospheric soil isolates	Six-component rhizospheric consortium
T8	Distilled water	No inoculum	Non-inoculated distilled-water control

Notes: Treatment codes T1–T8 indicate the experimental inoculation treatments. The prefixes used in the isolate codes correspond to internal CINCAE source-tracking codes and should not be interpreted as independent taxonomic ranks: gt = sugarcane stem-associated *G. diazotrophicus* isolate, gr = sugarcane root-associated *G. diazotrophicus* isolate, gh = sugarcane leaf-associated *G. diazotrophicus* isolate, and gs = rhizospheric soil-associated *G. diazotrophicus* isolate. The numerical suffixes identify the original CINCAE isolate code or sampling line and should not be interpreted as dose, replicate number, or taxonomic rank. T8 corresponded to the non-inoculated distilled-water control. All inoculated treatments were standardized before application to approximately 1.35 × 10^8^ ± 0.21 × 10^8^ CFU mL^−1^ and applied through the same 1 h immersion route under identical greenhouse conditions.

**Table 3:** PCR markers used for experimental verification of the CINCAE-sequenced *Gluconacetobacter diazotrophicus* isolate.

Amplicon Size	Target Gene/Marker	Primer Forward 5′–3′	Primer Reverse 5′–3′	Specificity/Interpretive Scope	Analytical Purpose	Reference Support
218 bp	*G. diazotrophicus*-associated 16S–23S rDNA internal transcribed spacer/intergenic spacer region	5′-AGC TGG GTG GGG TAA AG-3′	5′-GTA CCG TCA TCA TCG TCC CC-3′	Species-associated marker used as experimental support for the CINCAE-confirmed *G. diazotrophicus* isolate.	Support for the presence of the target inoculum	[42]
~1491 bp	nearly full-length bacterial 16S rRNA gene	16S rRNA FC27: 5′-AGA GTT TGA TCC TGG CTC AG-3′	16S rRNA RC1492: 5′-TAC GGC TAC CTT GTT ACG ACT T-3′	Broad bacterial 16S rRNA marker; used together with the CINCAE sequencing report and species-associated marker	General bacterial identity support	[43,44,45]
395 bp	partial bacterial 16S rRNA gene fragment	16S rRNA F1114: 5′-GCA ACG AGC GCA ACC C-3′	16S rRNA RC1492: 5′-TAC GGC TAC CTT GTT ACG ACT T-3′	Partial bacterial 16S rRNA marker; not nifH; used as complementary internal bacterial support	Complementary bacterial identity support	[44,45]

## Data Availability

The original contributions presented in this study are included in the article. The CINCAE sequencing report supporting the taxonomic assignment of the isolate is available from the corresponding author upon reasonable request, subject to institutional data-sharing authorization.
